# 952 Global Characteristics and Outcomes of Massive Burn Injuries

**DOI:** 10.1093/jbcr/iraf019.483

**Published:** 2025-04-01

**Authors:** Daniel Najafali, Hilary Liu, Megan Najafali, Saeid Rezaei, José Arellano, Logan Galbraith, Mare Kaulakis, Erik Reiche, Victor Stams, Francesco Egro

**Affiliations:** Carle Illinois College of Medicine; University of Pittsburgh Medical Center; Loyola University Chicago Stritch School of Medicine; University of Pittsburgh Medical Center; University of Pittsburgh Medical Center; Northeast Ohio Medical University; University of Pittsburgh School of Medicine; University of Pittsburgh Medical Center; Carle Foundation Hospital; University of Pittsburgh Medical Center

## Abstract

**Introduction:**

Massive burn injuries represent a unique patient cohort requiring additional interventions and facing increased risk. Classification schemes of burn injuries focus on depth of injury and TBSA, but there is growing interest in distinguishing massive burns from other types of burn injuries. Given the need for different treatment strategies and higher care intensity, we aimed to characterize massive burns and quantify their impact on mortality.

**Methods:**

Patients were classified as having massive burns if their TBSA was 40% or greater. Descriptive statistics were used to give an overview of demographics and burn characteristics. Multivariable logistic regression analysis quantified the degree to which massive burns influence mortality. Secondary outcomes included the likelihood of undergoing surgical intervention and the presence of functional impairment at discharge among survivors.

**Results:**

There were 1,828 cases that met the criteria for massive burns. These patients had a median TBSA of 55% (IQR:45-75%). Almost half of the cohort sustained an inhalation injury (~48%) and the most common etiology of injury was flame-based (83%). Patients managed in low-resource regions accounted for 60% of massive burns. Mortality from massive burns was 68% and 22% survived to discharge. When controlling for other factors in multivariable regression analysis, massive burns increased odds of mortality (OR 7.25, 95%CI 6.08-8.65), decreased likelihood of undergoing surgical intervention (OR 0.55, 95%CI 0.48-0.62), and increased odds of functional impairment (OR 1.35, 95%CI 1.004-1.81).

**Conclusions:**

Massive burn injuries, defined as burns with a TBSA of 40% or greater, represent a high-risk population. The odds of death increased more than sevenfold, indicating that a massive burn is a significant predictor of mortality. These patients are less likely to undergo surgical intervention due to the severity of their condition. Survivors are more likely to experience long-term functional impairment, highlighting the need for comprehensive post-burn rehabilitation.

**Applicability of Research to Practice:**

Specific treatment protocols are needed for massive burns, especially in resource-limited regions. Establishing rehabilitation pathways is essential for improving long-term outcomes, particularly given the increased risk of functional impairment among survivors.

**Funding for the Study:**

N/A

